# Bypassing BamD essentiality by mutations in a non-essential substrate

**DOI:** 10.1128/mbio.01769-25

**Published:** 2025-08-18

**Authors:** Santosh Kumar, Anna Konovalova

**Affiliations:** 1 Department of Microbiology and Molecular Genetics, McGovern Medical School, The University of Texas Health Science Center at Houston (UTHealth) 12340, Houston, Texas, USA; National University of Singapore, Singapore, Singapore

**Keywords:** gram-negative bacteria, outer membrane biogenesis, Bam complex, protein folding

## Abstract

The β-barrel assembly machinery (Bam) is essential for assembling all transmembrane β-barrel outer membrane proteins in gram-negative bacteria. The Bam complex consists of the central β-barrel protein BamA and accessory Bam lipoproteins, including the widely conserved and essential BamD. BamD is assumed to be an essential regulator of OMP assembly, as its absence causes a global defect in OMP biogenesis. Here, we challenge this view by demonstrating that BamD essentiality is both conditional and substrate specific. In *Escherichia coli*, its function can be bypassed by preventing BamA jamming by a single, non-essential substrate RcsF. Our findings suggest that BamD plays two distinct roles in the Bam complex. It prevents improper RcsF engagement that can jam BamA, and it kinetically enhances BamA-mediated OMP assembly. Contrary to prevailing models, we demonstrate that the second function in general OMP assembly is not essential. We report a genetic background in which each Bam lipoprotein is dispensable for viability, providing a powerful new system for investigating their functions in OMP assembly in the context of unmodified, wild-type BamA.

**IMPORTANCE:**

The β-barrel assembly machinery (Bam) complex assembles all outer membrane proteins (OMPs) and is conserved and essential across all gram-negative bacteria. While BamA is critical for the folding and insertion of OMPs into the outer membrane, BamD is also considered essential for OMP assembly because its loss leads to a global reduction in OMP levels and cell death. Our results show that BamD is important but not essential for general OMP assembly. In *Escherichia coli,* BamD’s essentiality arises from its role in preventing a single challenging substrate from jamming BamA and indirectly abolishing OMP assembly. This work challenges long-standing assumptions about BamD’s function, underscores the substrate-specific roles of accessory Bam components, and offers important new considerations for interpreting genetic studies of the Bam complex.

## INTRODUCTION

Outer membrane (OM) biogenesis is an essential process in diderm bacteria. Transmembrane β-barrel OM proteins (OMPs) form a bulk of the OM, contributing to its mechanical stability and selective permeability (1, 2). OMPs also mediate nutrient uptake, envelope assembly and maintenance, and interaction with the environment.

The assembly of OMPs at the OM is performed by the β-barrel assembly machinery (Bam) (3, 4). The Bam complex consists of BamA (itself an OMP) and accessory Bam components (5, 6). It is widely acknowledged that BamA itself carries out the catalytic function of OMP folding and insertion into the OM. The β-barrel domain of BamA can laterally open to accommodate an incoming OMP substrate, facilitating its folding and lateral release into the OM (7–9). Much less progress has been made in understanding the molecular functions of Bam accessory components. In the model gram-negative bacterium *Escherichia coli*, there are four accessory lipoproteins, BamB–E. BamB, C, and E lipoproteins are individually not essential in *E. coli*. Their homologs are found in many but not all bacterial species, and there are also additional unique Bam components reported in other bacteria (10, 11). BamD is essential in *E. coli* and its deletion broadly affects OM biogenesis (12, 13), and, in contrast to other Bam lipoproteins, it is highly conserved in the Proteobacteria and is essential in many lineages (10–12, 14–17). As a result, BamD is believed to play a central and essential role in OMP assembly.

**Fig 1:**
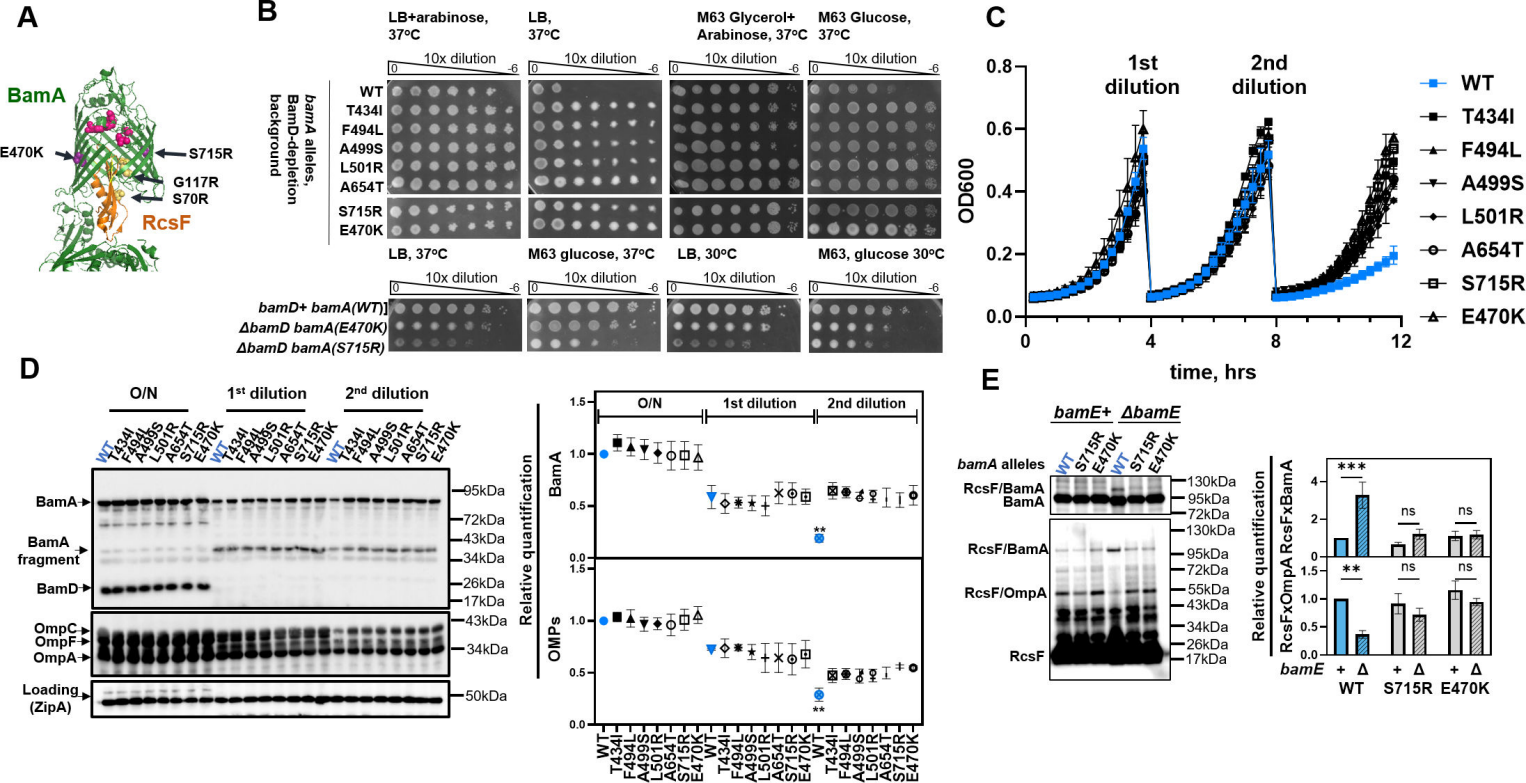
BamA variants that prevent RcsF jamming bypass BamD essentiality. (**A**) Mutant residues of BamA and RcsF that prevent jamming are shown as spheres on the structure of RcsF/BamA complex (PDB:6T1W) (28, 31). Select residues are labeled. (**B**) BamA variants restore growth under BamD depletion and, in some cases, in the Δ*bamD* background. Images are of an efficiency of plating (EOP) assay under the indicated growth conditions. (**C**) Growth curves of *bamA* mutants during BamD depletion (see SI Methods for description). Cultures were diluted twice to maintain growth, and samples were collected for immunoblotting at the time of dilutions. (**D**) Immunoblots and quantification of protein levels; statistical analysis by one-way ANOVA relative to WT under permissive conditions (mean ± SEM). BamA(WT) parent strain displayed statistically significant reduction in BamA and OMP levels at the time of second dilution compared to BamA mutant strains **P < 0.005. (**E**) BamA(E470K) prevents RcsF jamming, as assayed by *in vivo* crosslinking in the Δ*bamE* background. Figure represents immunoblots and relative quantification (mean ± SEM); statistical analysis by two-way ANOVA, significance for select pairs is indicated, **P < 0.005, ***P < 0.0005.

While the precise molecular function of BamD remains to be fully elucidated, previous genetic and biochemical analyses suggested that BamD plays a role in OMP substrate recruitment, regulation of BamA conformational cycling during OMP assembly, as well as the assembly of BamA itself (18–23). Our previous work uncovered that regulatory coordination between BamA and BamD is particularly important for the assembly of an interlocked complex between the lipoprotein RcsF and its partner OMPs (Fig. 1A) (24, 25), which function in the Rcs envelope stress response (26, 27). BamA/BamD coordination is supported by BamE, which enhances the overall Bam complex stability and provides an essential regulatory backup when direct interaction between BamA and BamD is compromised (24, 25). In *ΔbamE*, RcsF jams BamA by blocking the lumen of BamA’s β-barrel domain and its function in OMP assembly (Fig. 1B) (25, 28). As Δ*rscF* can rescue the growth of many synthetic lethal combinations of *ΔbamE* (25, 29, 30), we decided to investigate the relationship between RcsF and BamD. We report that when BamA jamming by RcsF is prevented, BamD is no longer essential. Contrary to the prevailing model, our results show that BamD is important but not essential for general OMP assembly. Considering the broad conservation of BamD, we propose that BamD’s essentiality arises from its role in preventing the improper engagement of challenging substrates with BamA.

## RESULTS

We have previously employed the *bamE bamB* synthetic lethality to isolate a collection of BamA and RcsF variants that prevent RcsF jamming to varying degrees (Fig. 1A) (28). Most *bamA* mutants prevented the jamming by destabilizing the BamA/RcsF complex, but our most potent *bamA(S715R*) mutant also restored RcsF/OMP assembly, thereby fully bypassing the BamE requirement. As our earlier work suggested that the underlying defect in *ΔbamE* is the inability of BamD to engage with BamA (24, 25), we investigated whether BamA mutant variants can bypass BamD regulation.

Because BamD is essential (12), we constructed BamD-depletion strains with a single copy of *bamA* expressed on a low-copy pZS21 plasmid. In this strain background, chromosomal expression of *bamD* is controlled by the P_BAD_ promoter, which can be induced by arabinose or tightly repressed by growth on glucose (32). To monitor growth, we first grew overnight (O/N) cultures in media containing arabinose, washed cells to remove arabinose, and grew serially diluted spot cultures under conditions of rapid growth (LB media) and slow growth (minimal media). As expected, BamD depletion strain with *bamA(WT*) allele failed to grow in the absence of arabinose (Fig. 1B). Surprisingly, our *bamA* mutant strains grew well on plates without arabinose, similar to *bamA(E470K*), which was previously reported to bypass BamD essentiality (33).

To monitor growth, Bam protein levels, and the efficiency of OMP assembly during BamD depletion, we used the approach developed by Tellez et al. (34). This approach capitalizes on the observation that cells can tolerate some decrease in Bam complex function under slow-growth conditions (minimal media). It allows strains that do not typically grow under standard conditions to survive for several generations, facilitating the assessment of strains for OMP assembly. In this approach, O/N cultures were grown in the minimal media supplemented with glycerol as a carbon source to initiate the removal of arabinose. This condition supports equal growth of all strains (Fig. S1) due to the substantial leaky expression of BamD from the P_BAD_ promoter (34). O/N cultures were diluted in minimal media with glucose as a carbon source to tightly repress *bamD* expression. At 4 and 8 h, cultures were diluted again in fresh media to maintain exponential growth (Fig. 1C).

We collected samples for immunoblotting from the O/N cultures and at the time of two subsequent dilutions (Fig. 1C and D). At the time of the first dilution, BamD was no longer detectable, but OMP and BamA levels remained unchanged, though we observed an accumulation of the BamA degradation fragment (Fig. 1D), which was reported to arise from the loss of BamA–BamD interactions (23). After the second dilution, *bamA(WT*) strain stopped growing (Fig. 1C), and the levels of all OMPs, including BamA itself, were severely reduced (Fig. 1D), indicating a severe OMP assembly defect. In contrast, *bamA* mutant strains demonstrated improved OMP assembly and grew normally (Fig. 1C and D).

BamA(E470K) can bypass *bamD* essentiality even in the presence of RcsF (33); hence, we reasoned that this mutation should also prevent RcsF jamming. Indeed, when we performed biochemical crosslinking in the *ΔbamE* background, we observed a reduction of the RcsF/BamA complex and the restoration of RcsF/OmpA, similar to our BamA(S715R) variant (Fig. 1E). Due to the similarity of the phenotypes, we reasoned that S715R may also be able to tolerate *ΔbamD* mutation. Indeed, we could delete *bamD* in this background when grown on M63 glucose media at 30°C without acquiring any suppressor mutations, as confirmed by whole-genome sequencing. We then assayed for growth under different conditions (Fig. 1B, lower panel). In summary, our results demonstrated that *bamA* mutations that prevent RcsF jamming allow BamD bypass and vice versa.

**Fig 2:**
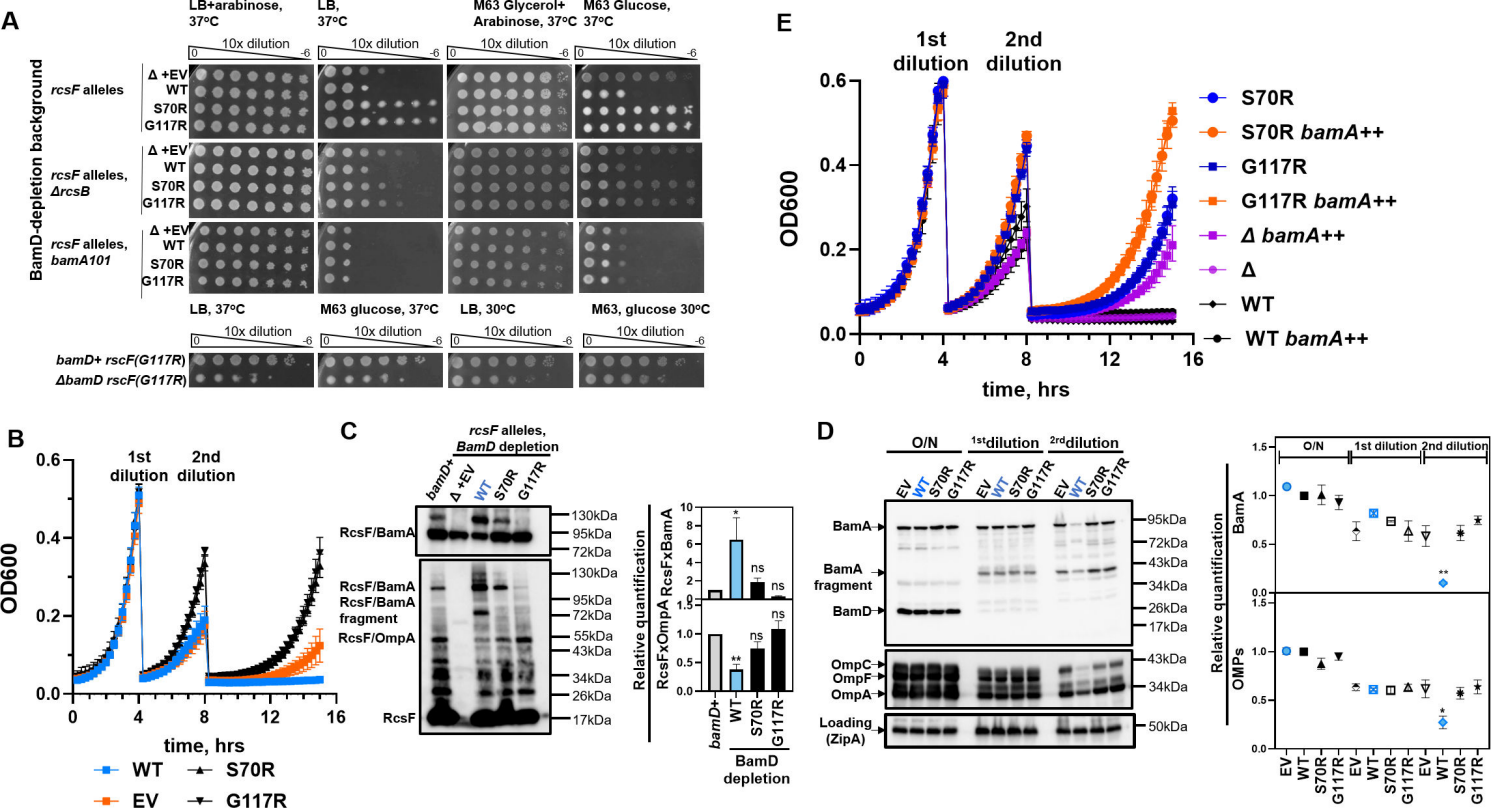
RcsF variants that prevent BamA jamming bypass BamD essentiality. (**A**) RcsF variants restore growth during BamD depletion and in the Δ*bamD* background, but require a functional Rcs stress response and normal BamA expression. Shown are EOP assays under indicated conditions. (**B**) Growth curves of *rcsF* mutants during BamD depletion, as described in Fig. 1. (**C**) *rcsF* mutations prevent BamA jamming, as shown by *in vivo* crosslinking at the time of the first dilution. Figure represents immunoblots and relative quantification (mean ± SEM); statistical analysis by two-way ANOVA, significance for select pairs is indicated, *P < 0.05, **P < 0.005. (**D**) Immunoblots and quantification of protein levels; statistical analysis by one-way ANOVA relative to WT in O/N cultures (mean ± SEM). *rcsF*(WT) parent strain displayed statistically significant reduction in BamA and OMP levels at the time of second dilution compared to *rcsF* mutant strains, *P < 0.05**, *P* < 0.005. (**E**) Growth curves of *rcsF* mutants expressing additional *bamA* from the plasmid (*bamA++*) during BamD depletion.

Next, we focused on the *rcsF* mutants that prevent BamA jamming (28). We observed that *ΔrcsF* improved the growth of the BamD depletion strain in minimal media but not in LB (Fig. 2A). Because *ΔrcsF* inactivates the protective Rcs stress response (2), we reasoned that this seesaw effect can mask suppression potential. Therefore, we turned to two previously characterized *rcsF* mutants, S70R and G117R, that prevent BamA jamming while maintaining the RcsF/OMP assembly and Rcs signaling (28). When we introduced these mutants into the *bamD* depletion background, the resulting strains grew well even in LB but displayed a mucoid phenotype indicative of Rcs induction (Fig. 2A). As expected, S70R and G117R mutants lost their advantage and looked similar to *ΔrcsF* when the Rcs stress response was inactivated by deleting the *rcsB* gene (Fig. 2A), underscoring the importance of Rcs in maintaining envelope integrity and promoting cell survival under BamD-depletion conditions.

The crosslinking assay at the time of the first dilution during BamD depletion revealed that most of the BamA was crosslinked with RcsF(WT) (Fig. 2B and C). Non-jamming *rcsF* alleles suppressed this phenotype, with G117R being the most potent (Fig. 2C). After the second dilution, the *rcsF* WT strain stopped growing and displayed severe OMP assembly defects (Fig. 2B and D). In the absence of RcsF, or in the presence of non-jamming *rcsF* alleles, levels of BamA and OMPs greatly increased compared to the parent control but not to the levels observed under permissive conditions (Fig. 2B and D). Together, our results demonstrate that the severe OMP assembly defect observed during the acute BamD depletion in the parent strain was indeed caused by RcsF-dependent BamA jamming.

We then wondered whether strains with non-jamming *rcsF* alleles could tolerate a complete loss of *bamD*. We could obtain multiple independent *ΔbamD::Kan* transductants when selected on the minimal media at 30°C. However, the *rcsF(S70R) ΔbamD::Kan* strain was unstable and picked up additional mutations, mainly in *bamA*. In contrast, *rcsF(G117R*), which is a much more potent suppressor of the jamming phenotype (Fig. 2C), was stable and did not pick up any additional mutations, as assayed by whole-genome sequencing, and hence could tolerate the deletion of *bamD. rcsF(G117R*) Δ*bamD* was mucoid (Fig. 2A, lower panel), indicative of the outer membrane defect, and grew more slowly than its *bamD+* parent and *bamA(E70K*) Δ*bamD* (Fig. S2). Nonetheless, the strain was viable under all tested conditions (Fig. 2A, lower panel).

These results demonstrated that BamA retains enough activity to support growth even in the absence of BamD. Consistent with this hypothesis, BamD depletion strains were unable to tolerate the reduction of BamA levels due to the *bamA101* knockdown allele (Fig. 2A). Conversely, increasing the copy number of BamA by introducing the pZS21::*bamA* plasmid further improved growth (Fig. 2E).

**Fig 3:**
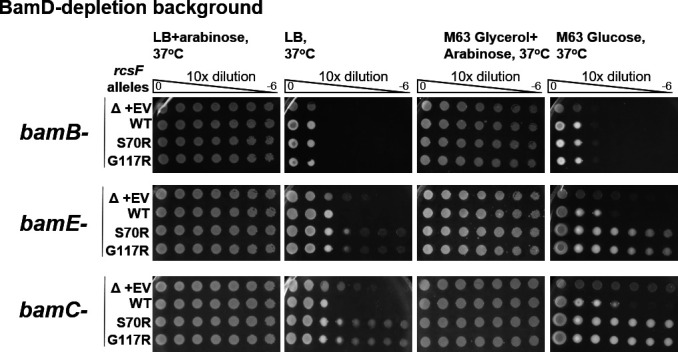
Growth of *rcsF* suppressor strains during BamD depletion requires BamB but not BamC or BamE activity. Shown are EOP assays under indicated conditions.

Non-jamming *rcsF* alleles provided a genetic background in which each Bam lipoprotein is dispensable for viability. Since we isolated these alleles in the screen for bypass of *bamB bamE* synthetic lethality (28), we investigated genetic interactions in the context of *bamD* depletion (Fig. 3). Deleting *bamE* or *bamC* in this background did not have any impact on growth, consistent with these components playing BamD-specific functions. Surprisingly, *bamB* became essential in this background, and strains were unable to grow even under the slow growth conditions (Fig. 3). This result suggests that, in the absence of RcsF jamming, either BamB or BamD is required for viability, highlighting their possible overlapping roles in general OMP assembly.

## DISCUSSION

We previously showed that disrupting the coordination between BamA and BamD, either through mutations at their interface or by deleting *bamE*, leads to the accumulation of RcsF on BamA (24, 25, 28). Our genetic and biochemical analyses demonstrated that the resulting RcsF/BamA complex is not a functional assembly intermediate but a dead-end complex that cannot dissociate (28). This results in BamA sequestration and, consequently, inhibition of OMP assembly. In this complex, RcsF plugs the lumen of the BamA barrel (28, 31). We isolated a set of *bamA* and *rcsF* mutations that destabilize this interaction *in vivo* and *in vitro* (28). In this study, we show that by relieving BamA jamming, these mutants render *bamD* non-essential.

On the basis of the results presented above, we propose that BamD serves two distinct functions within the Bam complex: (i) it prevents inappropriate engagement with RcsF that can jam BamA and (ii) it kinetically enhances OMP assembly by BamA. Contrary to the widely accepted model, the first function, not the second, is essential. This finding has important implications for interpreting genetic analyses of the Bam complex. Historically, the lack of viability of *bam* mutants has been attributed to a general loss of function of the Bam complex. Here, we report that the lack of viability can result from the defective assembly of a single, non-essential substrate, indirectly impairing overall OMP biogenesis and viability by sequestering BamA.

We demonstrate that mutations in *bamA* or *rcsF* that prevent BamA jamming can fully bypass BamD essentiality. Unlike *bamA* suppressors, which bypass BamD essentiality by modifying BamA in a way that is often difficult to study, the *rcsF* mutant background allows the bypass without altering the sequence of any of the Bam components. Hence, this mutant background provides a powerful new system for studying BamD’s role in general OMP assembly in the context of unmodified BamA and other Bam components. In the *rcsF(G117R*) background, depletion of BamD reduces growth and efficiency of OMP assembly compared to the normal level, suggesting that BamD kinetically enhances OMP assembly by BamA, and this defect can be overcome by increasing BamA levels.

Surprisingly, the survival of this strain was dependent on BamB, uncovering a potential overlapping role that both proteins play in OMP assembly. Interestingly, both proteins are implicated in the recruitment of OMP substrates to BamA. BamB, together with the Potra1 and Potra2 domains of BamA, facilitates the binding and regulation of SurA, the major periplasmic chaperone that delivers OMPs to the Bam complex (35–38). BamB was also proposed to improve OMPs delivery by promoting the formation of Bam supercomplexes (39). BamD, in contrast, interacts directly with unfolded OMPs and regulates BamA conformation to initiate OMP assembly (18, 19). However, we think it is also possible that BamD contributes to SurA-mediated OMP recruitment through its interaction with the Potra2 domain of BamA, a hypothesis supported by recent structural studies of Bam/SurA complexes (35, 36). As such, the simultaneous loss of BamD and BamB may block the recruitment of incoming OMPs.

The case study of RcsF provides an example of a substrate-dependent requirement for different Bam subunits. The lack of BamB mainly affects highly abundant substrates but has no effect on the assembly of more complex, slow-folding substrates, like essential LptD (40–42). In the *rcsF(G117R*) background, OMP assembly efficiency is reduced in the absence of BamD, as observed using model OMPs. However, the deletion of *bamD* may impact different OMP substrates to varying degrees. It is likely that BamD plays a more critical role in the assembly of specific, potentially more structurally complex substrates, though its function would need to be carefully (re)examined in the *rcsF(G117R*) background. Nonetheless, the synthetic lethal interaction between *bamD* and *bamB* demonstrates that they can at least partially compensate for one another.

RcsF remains the only known native substrate that can lethally jam BamA when misassembled (25, 29). Its proper assembly also uniquely depends on BamE to support BamD function (24, 25), highlighting the RcsF/OMP complex as particularly challenging to assemble. The broad conservation and essentiality of BamD, along with the notable conservation of BamE across gram-negative bacteria, suggest that other similarly challenging substrates, possibly other lipoprotein-OMP complexes, may exist and require distinct, substrate-specific roles for BamD and BamE in their assembly.

Our findings underscore the importance of mechanistic studies of individual OMP substrates, which vary greatly in size and domain architecture (43). We propose that this diversity necessitates additional accessory components that assist BamA in the efficient and accurate assembly of specific substrates.

## MATERIALS AND METHODS

Details about the media, growth conditions, strains used in this study, and strain construction are provided in the SI Materials and Methods.

### BamD depletion experiments in liquid cultures

O/N cultures were grown in M63 minimal media with glycerol as a carbon source. O/N cultures were diluted 1:100 in M63 media with glucose as a carbon source to repress *bamD* expression tightly. Cells were grown in 1 mL volume in a 24-well plate sealed with Breathe-Easy membrane (Sigma-Aldrich) at 37°C with orbital shaking in a BioTek Synergy H1 plate reader, and OD600 was monitored. At 4 and 8 h, cultures were diluted 1:50 in fresh M63 glucose media to maintain exponential growth. At the time of dilutions, cells from the remaining cultures were harvested, normalized by OD600, and subjected to immunoblot analysis. For crosslinking experiments during BamD depletion, cultures were grown as described above in 7 mL volume, and cells were harvested after 4 h. *In vivo* formaldehyde crosslinking was performed according to (25) and is described in detail in SI Materials and Methods.

Immunoblots were visualized using the ChemiDoc MP Imaging System (Bio-Rad), and quantified using Image Lab (Bio-Rad). Details on immunoblot procedures, antibodies, and quantification are provided in the SI Materials and Methods.

All experiments were performed in at least three independent biological replicates. Graph plotting and statistical analysis were performed by GraphPad Prism.

## SUPPLEMENTAL MATERIAL

Supplemental Material mbio.01769-25-s0001.docx Supporting Materials and Methods and figures.

ASM does not own the copyrights to Supplemental Material that may be linked to, or accessed through, an article. The authors have granted ASM a non-exclusive, world-wide license to publish the Supplemental Material files. Please contact the corresponding author directly for reuse.
