# Supplementary material for: Bypassing BamD essentiality by mutations in a non-essential substrate
Source: mBio. 2025 Aug 18;16(9):e01769-25. doi: 10.1128/mbio.01769-25 (PMC12421809; doi:10.1128/mbio.01769-25)
Supplement: Supplemental Material — Supporting Materials and Methods and figures. [file mbio.01769-25-s0001.docx]

**Supporting Information for**

Bypassing BamD essentiality by mutations in a non-essential substrate

Santosh Kumar and Anna Konovalova*.

* correspondence to Anna Konovalova.

**Email:**  anna.konovalova@uth.tmc.edu

**This PDF file includes:**

Supporting Materials and Methods

Supporting Figures

Supporting References

Supporting Materials and Methods.

Bacterial strains and growth conditions.

All the bacterial strains used in this study are listed below and derived from MC4100. Strains were grown at either 37°C or 30°C as indicated in Lysogeny broth (LB) [10 g/L tryptone, 5 g/L yeast extract, 10 g/L NaCl] or M63 medium [1x M63 salts (bioWorld), 0.1mg/mL thiamine, 1mM MgSO4, 0.1%casamino acid] with either glucose and glycerol at 0.2% as a carbon source. When necessary, L-arabinose was added to a final concentration of 0.2%; antibiotics were added when appropriate at the following concentrations: chloramphenicol 20 μg/mL, kanamycin 25 μg/mL, tetracycline 10 μg/ml, ampicillin 125 μg/mL. For strains SK287-292, media was supplemented with 2ng/ml anhydrotetracycline (Alfa Cesar) to ensure BamA expression.

All strains were constructed using standard microbiological techniques. For the construction of *ΔbamD::Kan*, P1 phage was raised on the SK-405 strain, and transductions were performed three independent times at 30^o^ C and recovered on M63 agar plates with glucose and kanamycin. The resulting colonies were streaked, and *bamD* deletion was confirmed by colony PCR with external and internal primers (BamD (in)F CAAAAGCTGCAGGACGGTAAC, BamD (in)R TGTATTGCTGCTGTTTGCGG, BamD (out)F FGCTTTGCCGTTTAATATAGTGTG, BamD (out)R GATGAGATCGATAGCGACTAAATC) and *bamA* gene was amplified and sequenced. The positive clones were propagated in M63 with glucose at 30^o^ C for all subsequent experiments. At least two clones from independent transductions were phenotypically characterized, and frozen at -80^o^ C. Cells from the same cultures that were used to generate -80^o^ C glycerol stocks were harvested and whole-genome sequenced (WGS) by SeqCenter LLC. Variant detection analysis was done in comparison to the corresponding genetic parent to ensure that no suppressor mutations arose during the strain construction.

**Strain Description**

pZS21::Kan (Lutz and Bujard 1997)

pZS21::Cm (Tata et al. 2021)

AK265 MC4100 *araR*/-  *PrprA-lacZ* (Konovalova, 2016)

JCM320 MC4100 *araR*/-; *ΔbamA* *λatt* (P_BAD_*-bamA, bla')* (Wu et al. 2005)

JCM290 MC4100 *araR*/-; *ΔbamD* *λatt* (P_BAD_*-bamD, bla')* (Malinverni et al. 2006)

AK1760 JCM290 *ΔrcsF*

SK234 JCM290 *ΔrcsF* //pZS21

SK235 JCM290 *ΔrcsF* //pZS21::rcsF(WT)

SK236 JCM290 *ΔrcsF* //pZS21::rcsF (S70R)

SK237 JCM290 *ΔrcsF* //pZS21::rcsF (G117R)

AK1518 JCM290//pZS21-twinstrep-*bamA*(WT)

AK1519 JCM290//pZS21-twinstrep-*bamA*(T434I)

AK1520 JCM290//pZS21-twinstrep-*bamA*(F494L)

AK1521 JCM290//pZS21-twinstrep-*bamA*(A499S)

AK1522 JCM290//pZS21-twinstrep-*bamA*(L501R)

AK1523 JCM290//pZS21-twinstrep-*bamA*(A654T)

AK1524 JCM290//pZS21-twinstrep-*bamA*(S715R)

SK281 JCM290//pZS21-twinstrep-*BamA*(E470K)

SK273 JCM290 *ΔbamA::cmR* // pZS21-twinstrep-*bamA(WT)*

SK274 JCM290 *ΔbamA::cmR* // pZS21-twinstrep-*bamA*(T434I)

SK275 JCM290 *ΔbamA::cmR* // pZS21-twinstrep-*bamA(F494L)*

SK276 JCM290 *ΔbamA::cmR* // pZS21-twinstrep-*bamA(A499S)*

SK277 JCM290 *ΔbamA::cmR* // pZS21-twinstrep-*bamA*(L501R)

SK278 JCM290 *ΔbamA::cmR* // pZS21-twinstrep-*bamA*(A654T)

SK279 JCM290 *ΔbamA::cmR* // pZS21-twinstrep-*bamA*(S715R)

SK284 JCM290 *ΔbamA::cmR* // pZS21-twinstrep-*bamA*(E470K)

MT382 *JCM320 nadB::tn10* (Tata et al. 2021)

MT404 *JCM320 ΔbamE nadB::tn10* (Tata et al. 2021)

SK287 *MT382/pZS21-twinstrep-bamA(WT)*

SK288 *MT382/pZS21-twinstrep-bamA(S715R)*

SK289 *MT382/pZS21-twinstrep-bamA(E470K)*

SK290 *MT404/pZS21-twinstrep-bamA(WT)*

SK291 *MT404/pZS21-twinstrep-bamA(S715R)*

SK292 *MT404/pZS21-twinstrep-bamA(E470K)*

SK320 *ΔrcsF PrprA-lacZ* // pZS21::*rcsF(G117R)*

SK324 *JCM320 ΔrcsF* // pZS21

SK325 *JCM320 ΔrcsF* // pZS21::rcsF(WT)

SK326 *JCM320 ΔrcsF* // pZS21::rcsF(S70R)

SK327 *JCM320 ΔrcsF* // pZS21::rcsF(G117R)

SK337 JCM290 *ΔrcsF* // pZS21(*cmR)* (EV)

SK338 JCM290 *ΔrcsF* // pZS21(*cmR)* rcsF(WT)

SK339 JCM290 *ΔrcsF* // pZS21(*cmR)* rcsF(S70R)

SK340 JCM290 *ΔrcsF* // pZS21(*cmR)* rcsF(G117R)

SK342 JCM290 *ΔrcsF ΔrcsB*

SK344 JCM290 *ΔrcsF ΔrcsB* // pZS21(EV)

SK345 JCM290 *ΔrcsF ΔrcsB* // pZS21::rcsF(WT)

SK346 JCM290 *ΔrcsF ΔrcsB* // pZS21::rcsF(S70R)

SK347 JCM290 *ΔrcsF ΔrcsB* // pZS21::rcsF(G117R)

SK357 JCM290 *ΔrcsF bamA101* // pZS21(*cmR)*

SK358 JCM290 *ΔrcsF bamA101* // pZS21::*rcsF*(WT) (CamR)

SK359 JCM290 *ΔrcsF bamA101* // pZS21(*cmR)* :: *rcsF*(S70R)

SK360 JCM290 *ΔrcsF bamA101* // pZS21(*cmR)* :: *rcsF* (G117R)

BH2308 *ΔbamA* attTn7::*bamA-E470K ΔbamD::kan* (Hart et al. 2020)

SK405 BH2308 // pTrc99::bamD

AK1438 *ΔrcsF yafC::Tn10 PrprA-lacZ* (Tata et al. 2021)

AK1437 *rcsF WT yafC::Tn10 PrprA-lacZ* (Tata et al. 2021)

AK1426 *rcsF S70R yafC::Tn10 PrprA-lacZ* (Tata et al. 2021)

AK1433 *rcsF G117R yafC::Tn10 PrprA-lacZ* (Tata et al. 2021), **WGS**

SK368 JCM290/pZS21:Cm

SK369 JCM290/pZS21:Cm/BamA

SK370 JCM290  *ΔrcsF*::Kan yafC::Tn10/ pZS21(*cmR)*

SK371 JCM290  *ΔrcsF* ::Kan yafC::Tn10// pZS21(*cmR)::*bamA

SK372 JCM290 rcsF(S70R) yafC::Tn10/ pZS21(*cmR)*

SK373 JCM290 *rcsF(S70R)* yafC::Tn10 // pZS21(*cmR)::b*amA

SK374 JCM290 *rcsF(G117R)* yafC::Tn10// pZS21(*cmR)*

SK375 JCM290 *rcsF(G117R)* yafC::Tn10/ pZS21(*cmR)::b*amA

SK401 JCM290 *ΔrcsF* // pZs21::Kan

SK411 *ΔrcsF yafC::Tn10 PrprA-lacZ* // pZS21(*cmR)*

SK412 *ΔrcsF yafC::Tn10 PrprA-lacZ* // pZS21(*cmR)* ::*rcsF*(WT)

SK413 *ΔrcsF yafC::Tn10 PrprA-lacZ* // pZS21(*cmR)* ::*rcsF*(S70R)

SK414 *ΔrcsF yafC::Tn10 PrprA-lacZ* // pZS21(*cmR)* ::*rcsF*(G117R)

SK454 *ΔrcsF yafC::Tn10 PrprA-lacZ Δbam*D::Kan // pZS21(*cmR)*::*rcsF*(G117R), **WGS**

SK471 *rcsF(G117R)* *yafC*::Tn10 *PrprA-lacZ, Δbam*D::Kan, **WGS**

SK489 AK-265 *ΔbamA* //pZS21(cmR)::*bamA(S715R),* **WGS**

SK490 AK-265 *ΔbamA* //pZS21(cmR)::*bamA(E470K),* **WGS**

SK491 *ΔbamA Δbam*D::Kan *PrprA-lacZ* // pZS21(*cmR)*::*bamA*(E470K) *,* **WGS**

SK494 *ΔbamA*  *Δbam*D::Kan *PrprA-lacZ* // pZS21(*cmR)* ::*bamA*(S715R) , **WGS**

Efficiency of Plating Assay.

BamD-depletion strains were grown overnight in LB supplemented with arabinose at 37^o^ C. Cells were harvested and washed to remove arabinose prior to serial dilutions. All *ΔbamD::Kan* strains were grown in M63 with glucose at 30^o^ C, and normalized to equal OD600 prior to serial dilutions. Cell cultures were 10 x serially diluted into LB medium or M63 medium. Cells were spotted onto the indicated agar plates and incubated at 30° C and 37° C overnight.

*In vivo* formaldehyde crosslinking

For crosslinking experiments during BamD-depletion, cultures were grown as described above in 7 ml volume, and cells were harvested after 4 hours. Cells were harvested by centrifugation, washed twice in phosphate-buffered saline (PBS) (Na_2_HPO_4_ 10 mM, KH_2_PO_4_ 1.8 mM, KCl 2.7 mM, NaCl 137 mM, pH 7.6) and normalized to an optical density (OD_600_) of 10 in PBS. Crosslinking was carried out in 200 µL of cell suspension by the addition of formaldehyde to a final concentration of 0.7 % for 12 min at room temperature. The reaction was stopped by the addition of Tris-Cl (pH 6.8) to a final concentration of 100 mM. The cells were harvested by centrifugation and resuspended in 100 µl of BBB buffer (1x BugBuster Reagent (Millipore Sigma), 50 mM Tris-Cl, pH 6.8 and 1 µL Benzonase (Millipore Sigma)). After incubating on the bench for 2-3 mins, 100 µl of 2X SDS loading buffer was added and samples were heated at 65°C for 15 min, and used for immunoblotting.

SDS-PAGE, immunoblotting and quantification.

10 µL of OD600 normalized samples were separated on SDS-PAGE: 10% gel for BamA and BamD immunoblots, 10% gel with 8M urea for OMP immunoblots, and 10% Tricine gel for RcsF crosslinking immunoblots. The proteins were blotted onto a Polyvinylidene difluoride (PVDF) membrane and blocked with 2 % non-fat dried milk in Wash buffer (1.21 g/L Tris base, 9 g/L NaCL, 0.05% Tween-20). The membranes were probed with polyclonal rabbit antibodies previously validated for specificity: anti- RcsF (1:10,000) (Konovalova, Mitchell, and Silhavy 2016), anti-BamA (1:40,000) (Wu et al. 2005), anti-OmpA, OmpC and OmpF (1:20,000) (Misra and Reeves 1987; Zimmermann and Wickner 1983), anti-BamD (1:5,000) (Sklar et al. 2007). Equal loading was confirmed by reprobing BamA and BamD blots with antibodies against ZipA (1:50,000)(Geissler, Elraheb, and Margolin 2003), an essential cytoplasmic protein. Donkey anti-rabbit IgG linked to HRP (1:10,000) (GE Healthcare) was used as a secondary antibody.

Digital measurements of chemiluminescence was performed using the ChemiDoc MP Imaging System (Bio-Rad) in the signal accumulation mode. Quantification analysis and blot visualization was performed using Image Lab (Bio-Rad) software. Relative band quantification was performed using O/N culture sample of the WT parent strain as a reference. Because OMPs are very close in molecular weight and do not separate well, the total signal of OMP bands was quantified. Figures show representative immunoblots. Quantification graphs represent mean +/-SEM of at least three biological replicates.

**Supplementary Figures.**


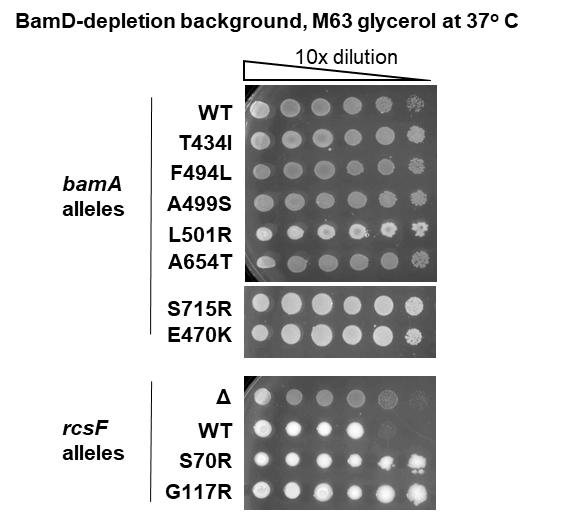


**Figure S1.** Viability of *bamA* and *rcsF* mutant strains in the BamD-depletion background during overnight growth in M63 glycerol media. Images are an efficiency of plating (EOP) assay under the indicated growth conditions.

**Figure S2**. Growth curves of *rcsF(G117R) ΔbamD* strain in comparison to the *bamD+* parent and previously characterized *bamA(E470K) ΔbamD* strain under indicated growth conditions.

**SI References**

Hart, E. M., M. Gupta, M. Wuhr, and T. J. Silhavy. 2020. 'The gain-of-function allele bamA(E470K) bypasses the essential requirement for BamD in beta-barrel outer membrane protein assembly', *Proc Natl Acad Sci U S A*, 117: 18737-43.

Lutz, R., and H. Bujard. 1997. 'Independent and tight regulation of transcriptional units in Escherichia coli via the LacR/O, the TetR/O and AraC/I1-I2 regulatory elements', *Nucleic Acids Res*, 25: 1203-10.

Malinverni, J. C., J. Werner, S. Kim, J. G. Sklar, D. Kahne, R. Misra, and T. J. Silhavy. 2006. 'YfiO stabilizes the YaeT complex and is essential for outer membrane protein assembly in Escherichia coli', *Mol Microbiol*, 61: 151-64.

Tata, M., and A. Konovalova. 2019. 'Improper Coordination of BamA and BamD Results in Bam Complex Jamming by a Lipoprotein Substrate', *mBio*, 10.

Tata, M., S. Kumar, S. R. Lach, S. Saha, E. M. Hart, and A. Konovalova. 2021. 'High-throughput suppressor screen demonstrates that RcsF monitors outer membrane integrity and not Bam complex function', *Proc Natl Acad Sci U S A*, 118.

Tellez, R., Jr., and R. Misra. 2012. 'Substitutions in the BamA beta-barrel domain overcome the conditional lethal phenotype of a DeltabamB DeltabamE strain of Escherichia coli', *J Bacteriol*, 194: 317-24.

Wu, T., J. Malinverni, N. Ruiz, S. Kim, T. J. Silhavy, and D. Kahne. 2005. 'Identification of a multicomponent complex required for outer membrane biogenesis in Escherichia coli', *Cell*, 121: 235-45.

Geissler, B., D. Elraheb, and W. Margolin. 2003. 'A gain-of-function mutation in ftsA bypasses the requirement for the essential cell division gene zipA in Escherichia coli', *Proc Natl Acad Sci U S A*, 100: 4197-202.

Konovalova, A., A. M. Mitchell, and T. J. Silhavy. 2016. 'A lipoprotein/beta-barrel complex monitors lipopolysaccharide integrity transducing information across the outer membrane', *Elife*, 5.

Misra, R., and P. R. Reeves. 1987. 'Role of micF in the tolC-mediated regulation of OmpF, a major outer membrane protein of *Escherichia coli* K-12', *J Bacteriol*, 169: 4722-30.

Sklar, J. G., T. Wu, L. S. Gronenberg, J. C. Malinverni, D. Kahne, and T. J. Silhavy. 2007. 'Lipoprotein SmpA is a component of the YaeT complex that assembles outer membrane proteins in Escherichia coli', *Proc Natl Acad Sci U S A*, 104: 6400-5.

Wu, T., J. Malinverni, N. Ruiz, S. Kim, T. J. Silhavy, and D. Kahne. 2005. 'Identification of a multicomponent complex required for outer membrane biogenesis in Escherichia coli', *Cell*, 121: 235-45.

Zimmermann, R., and W. Wickner. 1983. 'Energetics and intermediates of the assembly of Protein OmpA into the outer membrane of *Escherichia coli*', *J Biol Chem*, 258: 3920-5.
